# Acute effects of cigarette smoke on Endothelial Nitric Oxide synthase, vascular cell adhesion molecule 1 and aortic intima media thickness

**DOI:** 10.12688/f1000research.28375.4

**Published:** 2023-10-10

**Authors:** Meity Ardiana, Anwar Santoso, Hanestya Oky Hermawan, Ricardo Adrian Nugraha, Budi Susetyo Pikir, I. Gde Rurus Suryawan

**Affiliations:** 1 Department of Cardiology and Vascular Medicine, Faculty of Medicine Universitas Airlangga - Dr. Soetomo General Academic Hospital, Surabaya, East Java, 60272, Indonesia; 2 Department of Cardiology and Vascular Medicine, Faculty of Medicine University of Indonesia - National Cardiovascular Centre Harapan Kita Hospital, Jakarta, DKI Jakarta, 11420, Indonesia; 3 Department of Biomedicine, Faculty of Medicine, Universitas Brawijaya, Malang, East Java, 65145, Indonesia

**Keywords:** aortic tissue, atherosclerosis, cigarette smoking, endothelial-NOS, intima media thickness, VCAM-1

## Abstract

*Background.* Cigarette smoking could induce endothelial dysfunction and the increase of circulating markers of inflammation by activation of monocytes. This can lead to increased intima media thickness (IMT) of entire blood vessels and result in acceleration of the atherosclerosis process. However, to our knowledge, little is known about the role of cigarette smoking in this atherosclerotic inflammatory process.

The aim of this study is to explore the link between cigarette smoking and its effect on endothelial nitric oxide synthase (e-NOS) and vascular cell adhesion molecule 1 (VCAM-1).

*Methods.* An experimental study with a post-test only controlled group design was used. We used 18 Wistar rats ( *Rattus norvegicus*) randomly subdivided into two groups: group K (-) were not exposed to tobacco smoke, whereas group K (+) were exposed to smoke equivalent of more than 40 cigarettes for 28 days daily. After 28 days, samples were analyzed for e-NOS, VCAM-1 and aortic IMT.

*Results* *. *Our results indicate that tobacco smoke can enhance the expression of VCAM-1 on rat cardiac vascular endothelial cells, resulting in a decreased expression of e-NOS level and increase of aortic IMT. Linear regression model found that eNOS level negatively correlated wiith aortic IMT ( *r* ^2^ = 0.584, β = -0.764, *p* < 0.001), whereas VCAM-1 expression did not correlate with aortic IMT ( *r* ^2^ = 0.197, *p* = 0.065).

*Conclusion.* Low e-NOS level and high VCAM-1 level observed after cigarette smoke exposure which may increase aortic IMT.

## Abbreviations

ANOVA: analysis of variant

BH4: Tetrahydrobipterin

ELISA: enzyme-linked immunosorbent assay

e-NOS: endothelial Nitric Oxide Synthase

H _2_O _2_: Hydrogen peroxide

IACUC: Institutional Animal Care and Use Committee

IHC: Immunohistochemistry

IMT: Intima–media thickness

IRS: Immunoreactivity Scoring System

LSAB: Labeled Streptavidin Avidin Biotin

NIH: National Institutes of Health

PCR: Polymerase Chain Reaction

SA-HRP: Strepavidin-Hoseradish Peroxidase

SD: standard deviation

SEM: standard error of the mean

SPSS: Statistical Package for the Social Sciences

TGF: Transforming Growth Factor

VCAM-1: Vascular Cell Adhesion Molecule-1

### Clinical significance

Increasing evidence suggests that cigarette smoke exposure could induce VCAM-1 (enhance pro-atherogenic property), and a decrease of e-NOS level (anti-atherogenic depletioon). Thus, cigarette smoke may represent a significant risk factor for atherosclerosis by increasing aortic IMT. This evidence is discussed herein.

## Introduction

Cigarette smoking is the most important modifiable risk factor for developing atherosclerosis including cerebrovascular accident, peripheral arterial disease, and coronary heart disease. ^1^ In a meta-analysis from 55 eligible studies (43 cross-sectional, ten cohort and two case-control studies), the odds ratio (ORs) of peripheral arterial disease (PAD) associated with cigarette exposed was 2.71 (95% CI: 2.28-3.21; *p* < 0.001). ^2^ In a meta-analysis from 75 cohorts (2.4 million participants) that adjusted for cardiovascular risk factors other than coronary heart disease, multiple-adjusted pooled ORs of smoking versus non-smoking was 1.25 (95% CI: 1.12–1.39, *p* < 0.0001). ^3^

Even though epidemiologic studies clearly stated the negative effect of cigarette smoke for cardiovascular diseases, the underlying mechanisms have yet to be confirmed. The pathogenesis and pathophysiologic mechanisms by which exposure to cigarette smoke could accelerate atherosclerosis cardiovascular disease are complex and challenging, due to more than 5,000 different mixtures of chemicals inside the cigarette smoke itself. ^4^ Several potential contributing factors to atherogenesis inside cigarette smoke are (1) polycyclic aromatic hydrocarbons, (2) oxidizing agents, (3) particulate matter, and (4) nicotine. ^5^

One of the most important factor contributing for pro-atherogenic is nicotine, which has commonly been studied using cigarette smoke condensates. ^6^ In addition to its role as the habituating agent in tobacco, nicotine also accelerates atherosclerosis cardiovascular disease. There are several potential mechanisms of the pro-atherogenic effects of nicotine: (1) inducing endothelial dysfunction, (2) modifying lipid profile, (3) increasing inflammatory response, (4) inducing the release of catecholamines, which may increases heart rate and blood pressure, (5) increases platelet aggregability, (6) direct actions on the cellular elements participating in plaque formation, and (7) induces the proliferation and migration of vascular smooth muscle cells into the intima, mediated in part by TGFβ. ^7^ These pathomechanisms of nicotine could lead to the increase of intima media thickness of the entire blood vessel, leading to the greater risk of developing atherosclerosis. ^8^

To learn more about the pathomechanisms of the diseased endothelium, we need to study all the oxidizing, inflammatory, and thrombotic molecules which are not in an equilibrium state. In the model of atherosclerosis cardiovascular diseases, a pathological imbalance between prothrombotic and antithrombotic state, prooxidant and antioxidant state, and pro-inflammatory and anti-inflammatory state are observed. ^9^ Considerable evidence supports the importance of inflammation and hypercoagulability to promote atherogenic state. ^10^ There is abundant literature concerning the role of biomarkers of pathological imbalance in atherosclerosis.

Cell adhesion molecules are the essential pro-inflammatory and pro-atherogenic proteins that represent a hallmark of endothelial dysfunction and atherosclerosis. P-selectin, vascular cell adhesion molecule (VCAM)-1, intercellular adhesion molecule (ICAM)-1, and PECAM-1 were demonstrated to be involved in the formation of atherosclerosis plaque. ^11^ Beyond the other cell adhesion molecules, VCAM-1 plays as an important factor in neointima proliferation following nicotine-induced arterial injury, an area of research important for atherosclerosis cardiovascular diseases. ^12^ In the nicotine-induced arterial injury model, VCAM-1 expression is highly induced in the proliferation and migration of neointimal smooth muscle cells. ^13^

Previous studies showed that upregulation of endothelial nitric oxide synthase (e-NOS) expression and activity has its important role in the protection of endothelium. ^14– 16^ e-NOS could stimulate endothelium-dependent relaxation and protect against the development of VCAM-1-induced endothelial dysfunction. ^17^ However, to our knowledge, little is known about the role of cigarette smoke in this atherosclerotic inflammatory process. This study aims to explore the link between cigarette smoke on e-NOS and VCAM-1, which results in the development of aortic intima media thickness (IMT) of the experimental animals.

## Methods

### Ethics approval

This article was reported in line with the ARRIVE guidelines. Animal experimental study were conducted under the approval of the Institutional Animal Care and Use Committee of Universitas Airlangga (UNAIR), Surabaya, Indonesia (animal approval no: 2.KE.184.10.2019) under the name of Meity Ardiana as the Principal Investigator. All efforts were made to ameliorate any suffering of animals through using anaesthetic to euthanize the rats at the end of the experimental procedure.

### Animals

The present study used 18 male Wistar rats ( *Rattus novergicus*), eight weeks of age (average body weight 150-200 grams). The rats were adapted to their environment for seven days before the experiment start. They were nurtured at the Animal House at Faculty of Veterinary Medicine, Universitas Airlangga in polycarbonate cage, which measured 480 mm × 265 mm × 210 mm. Each cage had wood shavings on the floor, and contained three or four animals, which were marked for each subgroup. The rats were housed in microisolator cages and maintained in a constant room temperature ranging from 22°C to 25°C, with a 12-h light/12-h dark cycle, under artificially controlled ventilation, with a relative humidity ranging from 50% to 60%. The rats were fed a standard balanced rodent diet and water was provided *ad libitum.* The rats were chosen for each group by simple random sampling. For anaesthesia, our standard operating procedures used the combination of isoflurane and ether to make sure that nitric oxide levels weren’t altered significantly. For euthanasia, our standard operating procedures used intraperitoneal injection of sodium pentobarbital 200 mg/kg BW. Our procedures were approved by the animal research ethic committee in compliance with IACUC policy on the use of laboratory animal protocol. Study was carried out in strict accordance with internationally-accepted standards of the Guide for the Care and Use of Laboratory Animals of the National Institute of Health and in line with the ‘Animal Research: Reporting in vivo Experiments’ (ARRIVE) Guidelines. After that rats were euthanized, we collected the aortic tissue to obtain aortic intimal media tissue including isolation of rat aortic artery, removal of the fat tissue and branches, separation of longitudinal cutting edge, and peeling off the adventitia.

### Experimental design and groups

**Figure 1.**
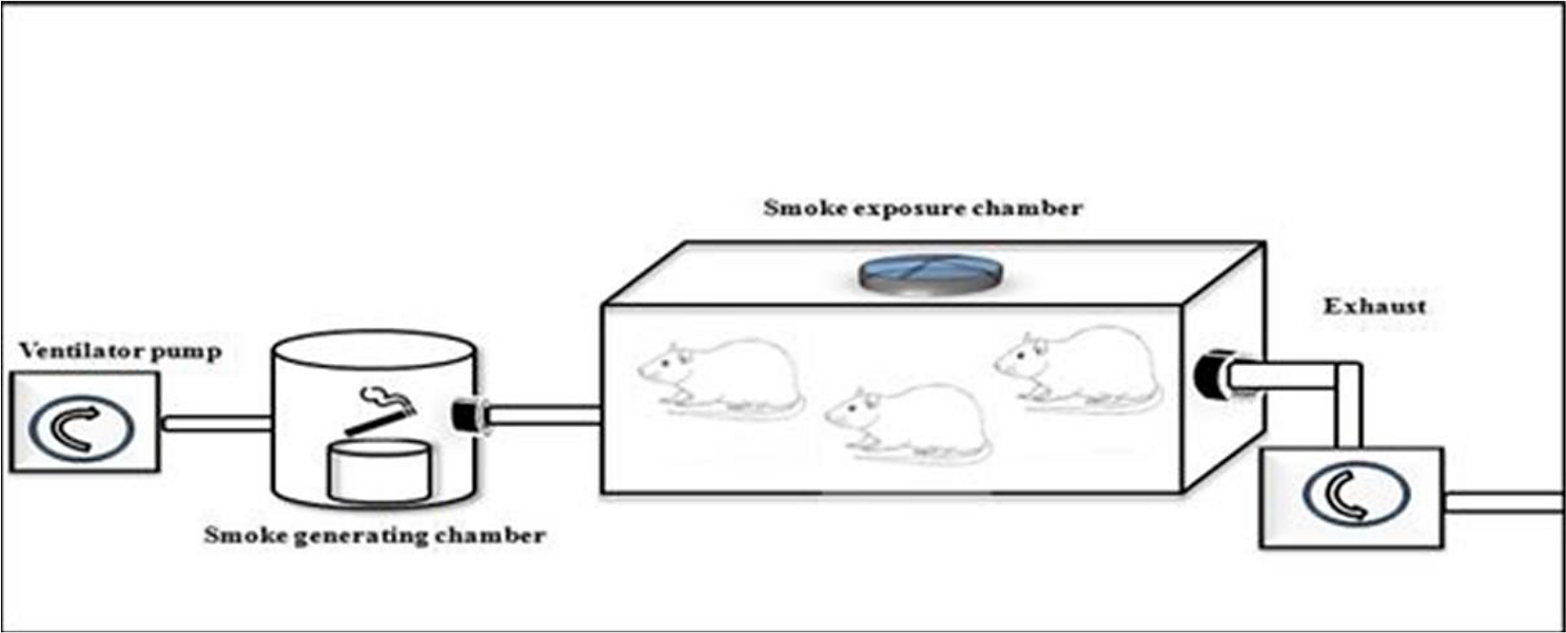
Illustration of how to exposed rats in the K(+) group to the cigarrete smokes. Exposure to tobacco smokes were done using side stream technique from peristaltic pump, smoke producer chamber, and inhalation chamber, connected by modified silicon tube.

The present study design was a randomized post-test only controlled group design using quantitative method. We extracted 18 male Wistar rats, randomized and then allocated them into two groups. Group 1 were not exposed to tobacco smoke, whilst group 2 were given 40 or more cigarette smokes daily for 28 days as seen in Figure 1. Each cigarette smoke dose contains 39 mg of tar and 2.3 mg of nicotine. The enrolled animals were analysed for vascular cell adhesion molecule 1 (VCAM-1), endothelial nitric oxide synthase (e-NOS), and aortic intima media thickness (IMT) after 28 days of consecutive experiments.

### Aortic Intima Media Thickness (IMT)

Thoracic aortas were prepared as distal aortic arch by cutting from the left ventricle of each rat. The post mortem samples of descending thoracic aortas obtained by dissection were fixed in 10% formaldehyde, embedded in paraffin, and sectioned at a thickness of 6 μm. The mounted tissues were stained using hematoxylin and eosin. Aortic intima media thickness was measured via Leica DMD 108 (Leica Microsystems GmbH, Wetzlar, Germany). Each sample was measured as “micrometer (μm)” from six different locations of the vessel wall. Arithmetic averages of these six measurements are presented in the results section.

### Vascular cell adhesion molecule 1 (VCAM-1)

We used streptavidin-biotin method which uses a biotin conjugated secondary antibody to link the primary antibody to a streptavidin-peroxidase complex for Immunohistochemistry (IHC) staining. The labeled streptavidin-biotin (LSAB) method were utilized to measure expresson of VCAM-1 in the aortic tissue of the rats. Firstly, the aortic tissue were prepared and preserved through deparaffinize models following fixation. Secondly, the aortic tissue was rehydrated by immersing the slides through the xylene (three washes, five minutes each), 100% ethanol (two washes, 10 minutes each), 95% ethanol (two washes, 10 minutes each), 70% ethanol (two washes, 10 minutes each), 50% ethanol (two washes, 10 minutes each), and deionized water (two washes, five minutes each). Thirdly, the aortic tissue was washed using Phosphat Buffer Sollution and then dipped into 3% of H _2_O _2_ solution withing 20 minutes. Fourthly, we added 1% of Bovine Serum Albumin to the Phosphat Buffer Sollution and then incubated them within 30 minutes in the room temperature. Fifthly, primary antibody anti-VCAM-1 (Santacruz biotech SC-13160) was added and incubated within 30 minutes, then washed again using Phosphat Buffer Sollution. Secondary antibody (Anti-Rat IgG Biotin Labelled) was added and incubated within 30 minutes in the room temperature, then washed using Phosphat Buffer Sollution. Sixthly, SA-HRP (Strepavidin-Hoseradish Peroxidase) complex was added and incubated within 10 minutes in the room temperature and then washed using Phosphat Buffer Sollution. Seventhly, Chromogen DAB (3,3-diaminobenzidine tetrahydrochloride) was added and incubated within 10 minutes in the room temperature, and then washed using Phosphat Buffer Sollution and sterile water. Finally, counterstain Hematoxylin-Eosin was added into the object glasses and expressions of VCAM-1 were measured and analyzed by a biological microscope (400× magnification) from tunica intima and tunica media of the aortic tissue. Semiquantitative measurements of VCAM-1 were done by immunoreactivity scoring system **(** Table 1 **)**.

**Table 1.** Immunoreactivity Scoring System (IRS).

Score for percentage of cells staining	Score for intensity of staining
0 = no stained cells 1 = <10% cells are stained 2 = 10-50% cells are stained 3 = 51-80% cells are stained 4 = >80% cells are stained	0 = no reaction 1 = mild intensity of staining 2 = moderate intensity of staining 3 = heavy intensity of staining

### Endothelial Nitric Oxide Synthase (e-NOS)

All samples were assessed by direct-sandwich enzyme-linked immunosorbent assay (ELISA) under the manufacture's system (R&D System Europe Ltd, Abingdon, UK) according to the National Institute for Biological Standards and Controls (Blanche Lane, South Mimms, Potters Bars, Hertfordshire, UK) protocol. We used eNOS kit from the elabscience (catalogue number: E-EL-R0367). Briefly, samples from the aortic tissue were collected and stored at −70 ^o^C (−94 ^o^F) at the Institute of Tropical Diseases Universitas Airlangga (UNAIR). Samples were homogenized into solution. Then, 100 μL of the solution was mixed with the well-coated primary antibody for e-NOS. Overnight incubation was done in a temperature of 4 ^o^C with a shaking machine for minimum 24 hours. Wash Buffer (20×) were diluted to 1× working solution with D.I. water prior to ELISA wash procedures. After that, 50 μL of the stop solution was added into each sample. A minimum value of 0.01 pg/mL were assigned for below the limit of detection.

### Statistical analysis

All measurements were performed and replicated at least three times. Results were presented as (1) means ± standard deviations (SD) for normally distributed data; (2) medians with lower and upper value for abnormally distributed data. The assumption of the normality for the complete data was assessed by Shapiro-Wilk test. Test of homogeneity of variances was assessed by Levene Statistics. Statistical significance were examined by Independent T-test, Mann-Whitney U test, and logistic regression using SPSS version 17.0 for Microsoft (IBM corp, Chicago, USA).

## Results

### Comparison of IMT level between smoke and non-smoke groups

**Figure 2.**
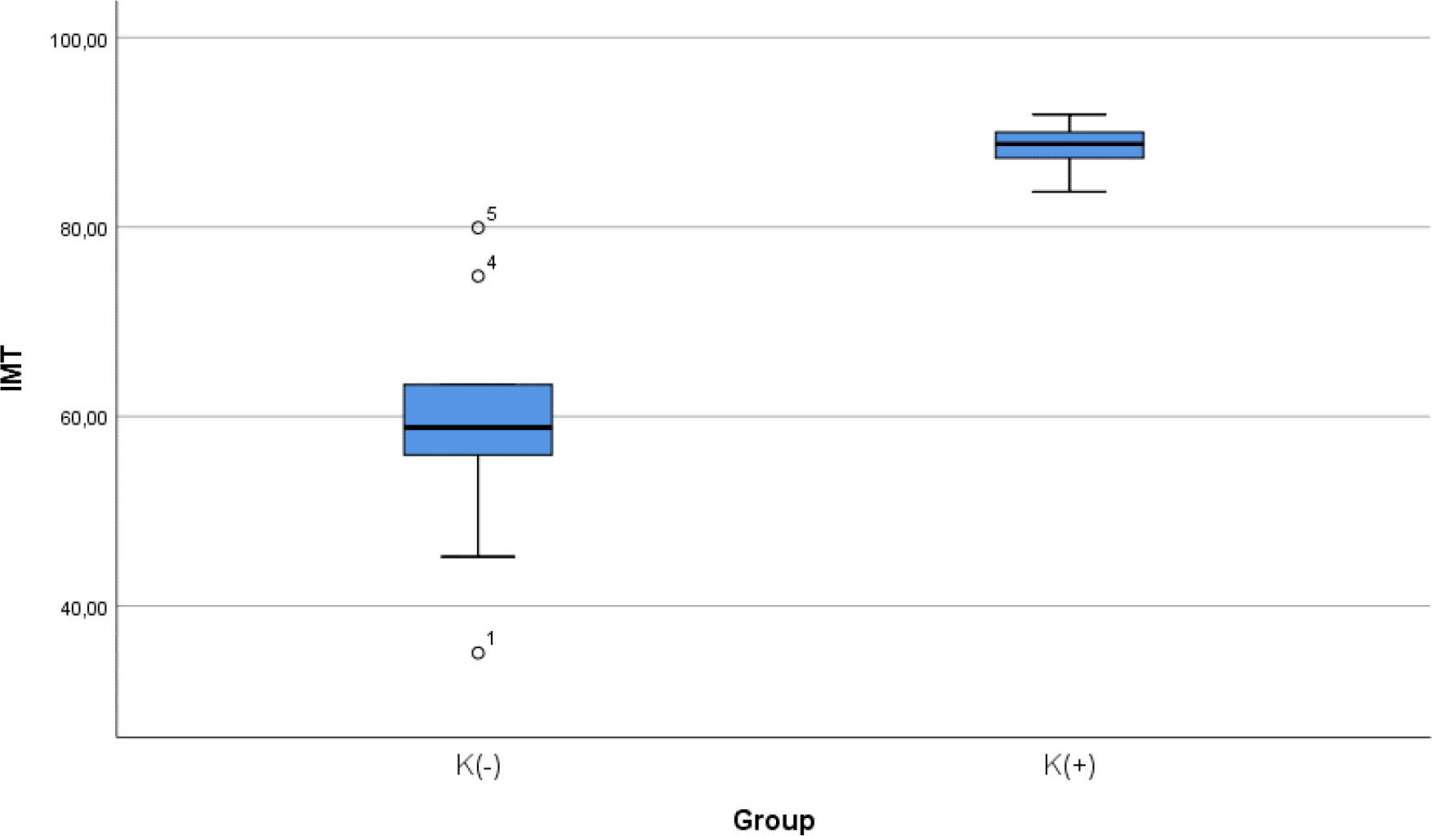
Median with lower and upper value of IMT between K(+) group which is exposed to the daily 40 cigarrete smokes and K(-) group as the control group.

After 28 days following experiments, there was a significance difference in IMT level between both groups ( *p* < 0.001). The mean of the aortic IMT in all animals were 73.68 ± 17.86 μm. The mean of the aortic IMT in the cigarette smoke group was 88.39 ± 2.51 μm. Thean of the aortic IMT in the control group was 58.98 ± 13.61 μm. Table 2 presents the impact of the exposure of smoke from 40 cigarettes daily on the aortic IMT profile of the experimental animals. The comparative analysis of IMT parameters demonstrated that there were statistically significant differences between the groups ( *p* < 0.001; Mann-Whitney’s test) ( Figure 2).

**Table 2.** Statistic table IMT between K(+) group which is exposed to the daily 40 cigarrete smokes and K(-) group as the control group.

Group statistics						
	Group	N	Mean	Std. Deviation	Std. Error Mean	Sig (Mann-Whitney)
IMT	K(-)	9	58.9800	13.61075	4.53692	<0.001
	K(+)	9	88.3911	2.51766	0.83922	

### Comparison of e-NOS level between smoke and non-smoke groups

**Figure 3.**
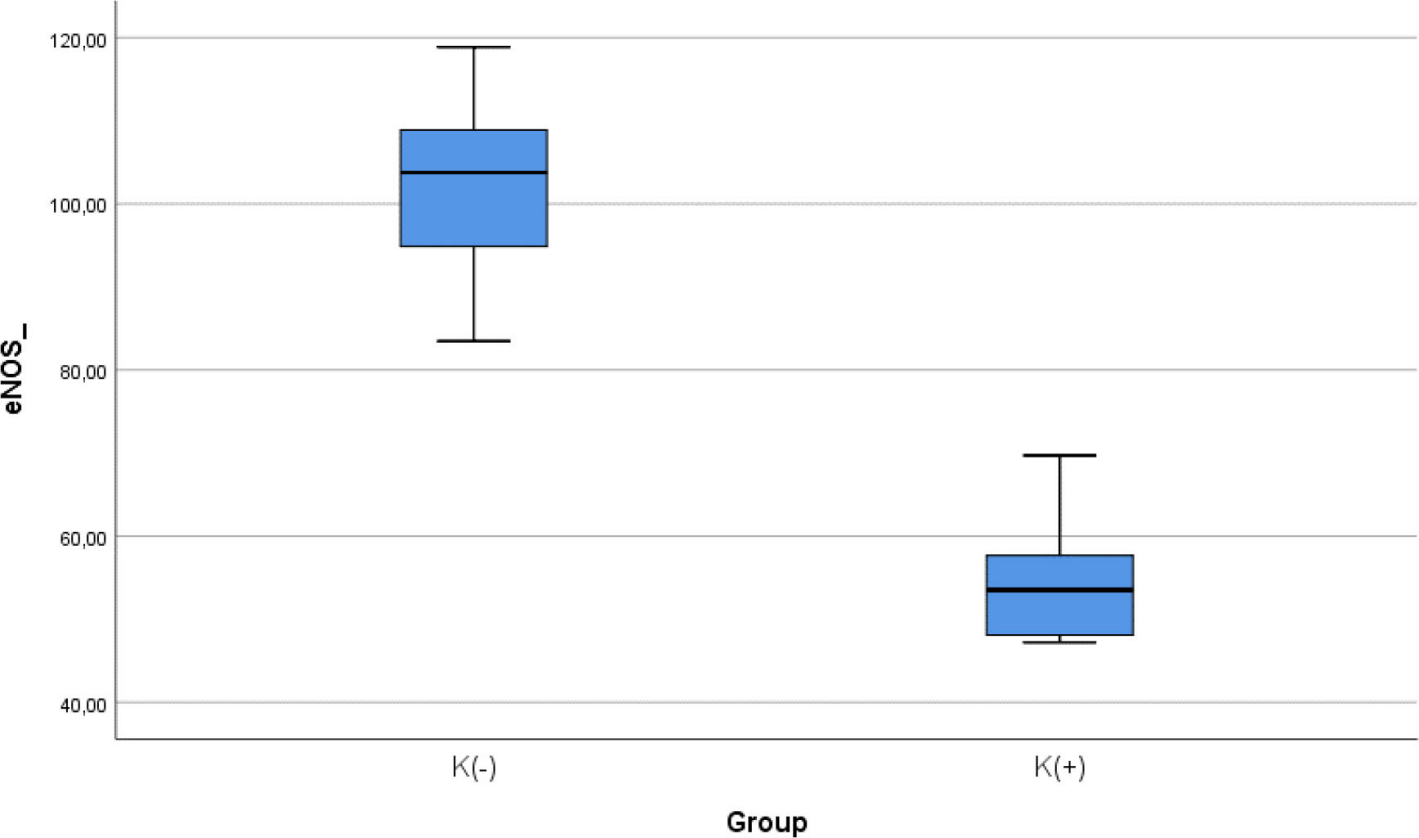
Median with lower and upper value of e-NOS between K(+) group which is exposed to the cigarrete smokes and K(-) group as the control group.

After 28 days following experiments, there was a significance difference of e-NOS level between both groups ( *p* < 0.001). The mean of the e-NOS in all subjects was 78.02 ± 25.84 pg/ml. The mean of the e-NOS level in the cigarette smoke group was 101.22 ± 11.8 pg/ml. The mean of the e-NOS level in the control group was 54.83 ± 8.3 pg/ml. Table 3 presents the impact of the exposure of daily smoke from the equivalent of 40 cigarettes on the e-NOS profile of the experimental animals. The comparative analysis of e-NOS parameters demonstrated that there were statistically significant differences between the groups ( *p* < 0.001; Mann-Whitney’s test) ( Figure 3).

**Table 3.** Statistic table e-NOS between K(+) group which is exposed to the cigarrete smokes and K(-) group as the control group.

Group statistics						
	Group	N	Mean	Std. Deviation	Std. Error Mean	Sig (Mann-Whitney)
eNOS_	K(-)	9	101.2233	11.80266	3.93422	<0.001
	K(+)	9	54.8267	8.30862	2.76954	

### Comparison of VCAM-1 expression between smoke and non-smoke

**Figure 4.**
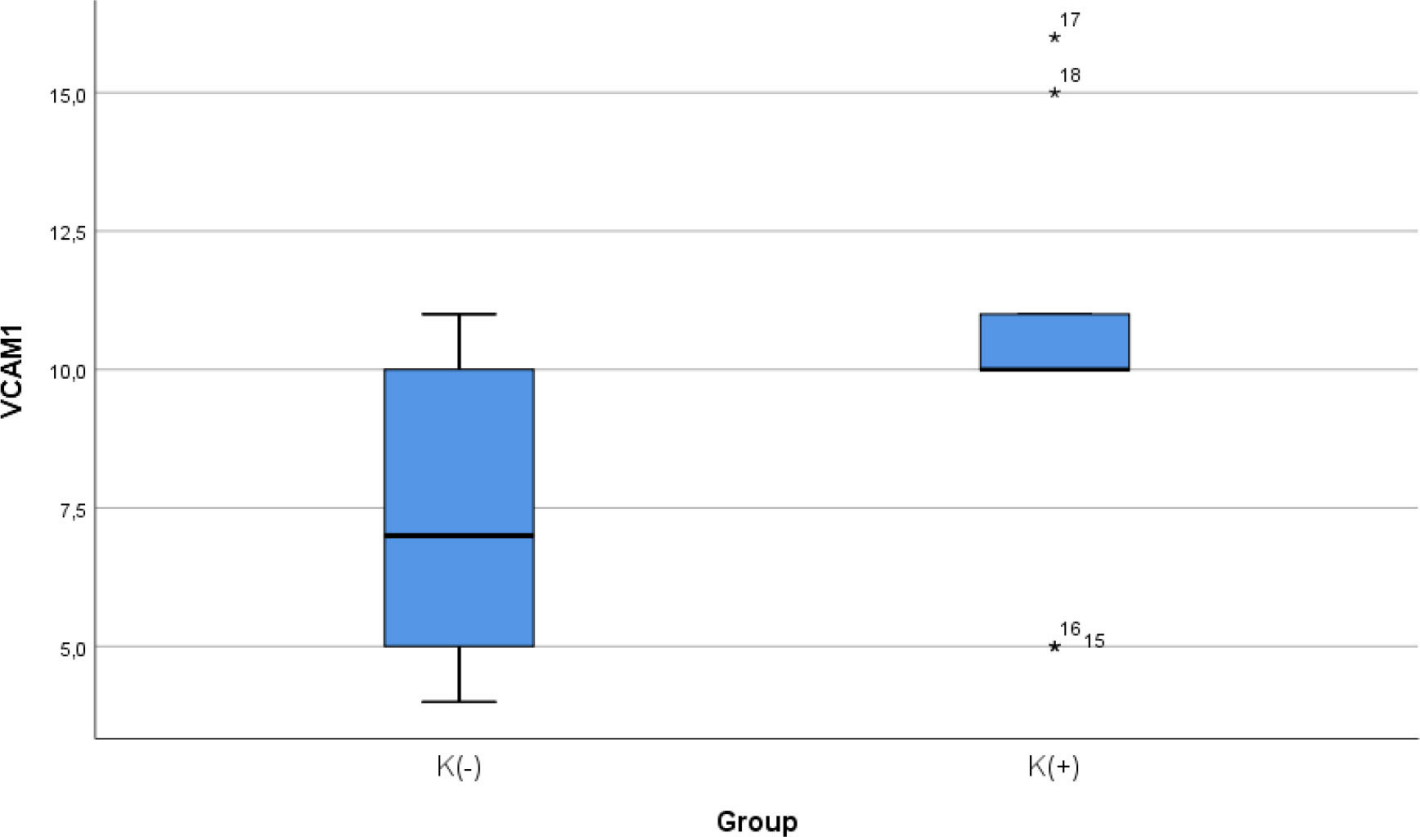
Median with lower and upper value of VCAM-1 between K(+) group which is exposed to the cigarrete smokes and K(-) group as the control group.

After 28 days following experiments, the mean of the VCAM-1 expression in all animals was 9.00 ± 3.51. The mean of the VCAM-1 level in the cigarette smoke group was 10.33 ± 2.9. The mean of the VCAM-1 level in the control group was 7.67 ± 3.7. Table 4 presents the impact of the exposure of daily 40 or more cigarette smokes on the VCAM-1 expression of the experimental animals. The comparative analysis of VCAM-1 expression demonstrated that there were no statistically significant differences between the groups ( *p* = 0.112; independent t test) ( Figure 4).

**Table 4.** Statistic table of VCAM-1 between K(+) group which is exposed to the cigarrete smokes and K(-) group as the control group.

Group statistics						
	Group	N	Mean	Std. Deviation	Std. Error Mean	Sig (Independent T)
VCAM1	K(-)	9	7.67	2.915	0.972	0.112
	K(+)	9	10.33	3.742	1.247	

### Correlation of e-NOS level and aortic IMT

**Figure 5.**
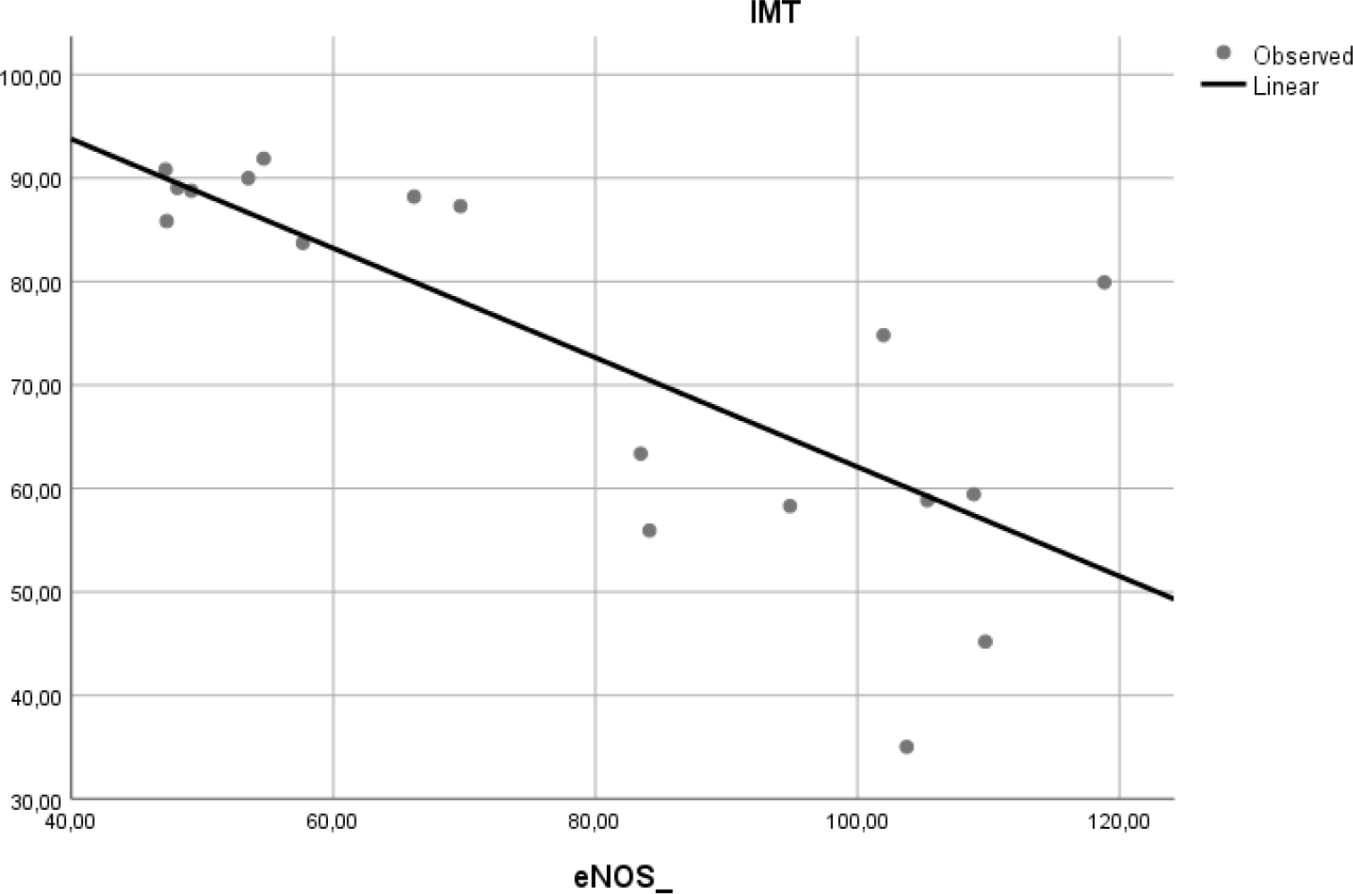
Relation between e-NOS level and aortic IMT in experimental rats. A negative linear relationship was found between e-NOS level and aortic IMT.

To determine if the level of e-NOS is correlated with atherosclerosis, we measured e-NOS as a parameter of endothelial cell function in aortic tissue of Wistar rats. Linear regression model found that e-NOS was negatively correlated with aortic IMT in our experimental study (r ^2^ = 0.584, β = −0.764, *p* < 0.001) ( Figure 5).

### Correlation of VCAM-1 expression and aortic IMT

**Figure 6.**
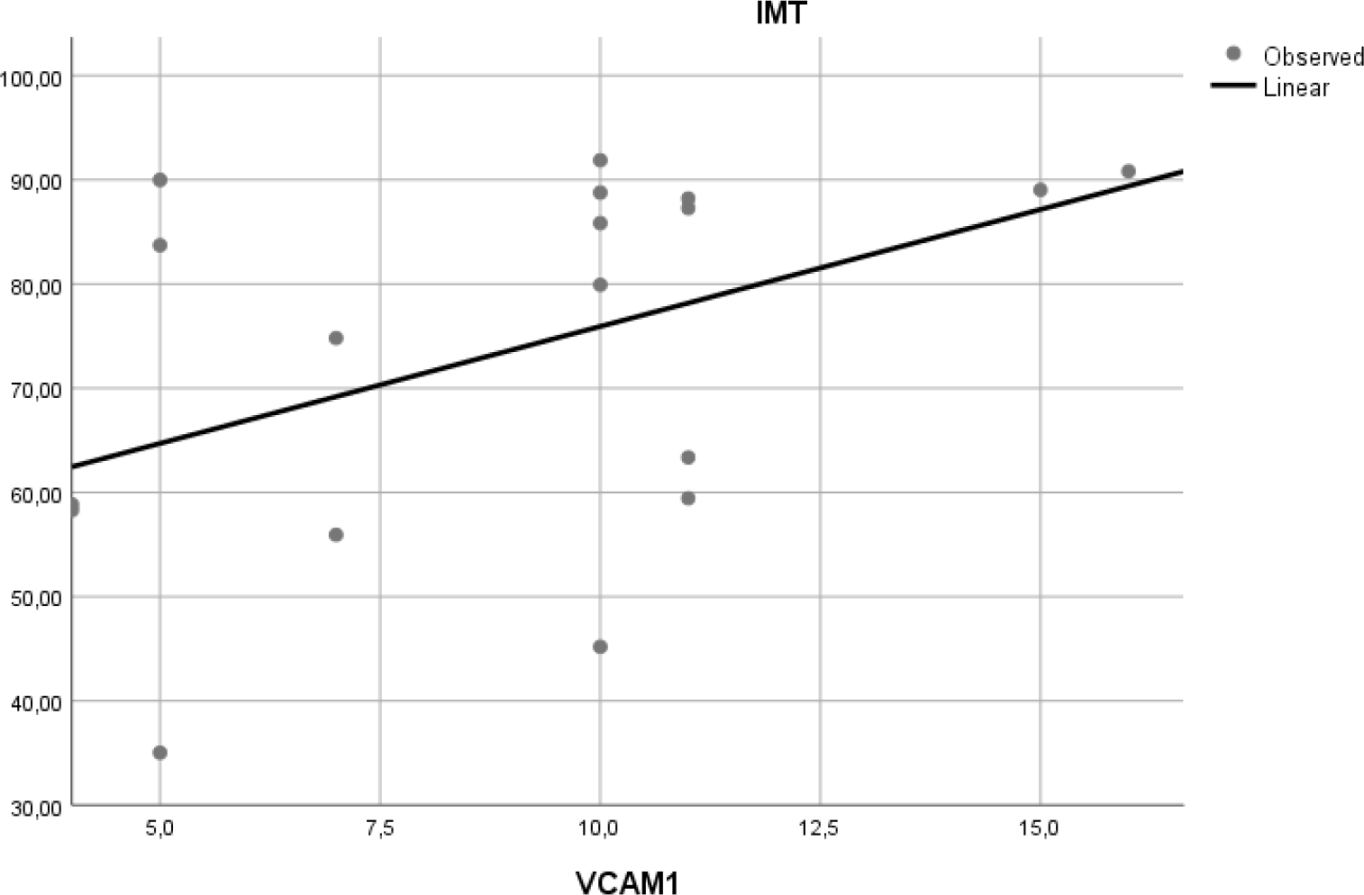
Relation between aortic VCAM-1 expression and aortic IMT in experimental rats. A positive but non-significant linear relationship was found between aortic VCAM-1 expression and aortic IMT.

To determine if expression of VCAM-1 precedes atherosclerosis, we measured expression of this adhesion molecule in aortic tissue of Wistar rats. Linear regression model found that VCAM-1 expression did not correlate with aortic IMT (r ^2^ = 0.197, *p* = 0.065) ( Figure 6).

## Discussion

### Oxidative stress-mediated cigarette smokes precedes atherosclerosis

Smoking cigarettes is one of the well-established modifiable risk factors for developing atherosclerosis, which mechanisms remain closely linked to the increased oxidative stress. The total amount of cigarettes smoked per day plays an essential role in increasing the level of oxidative stress and depletion of the antioxidant system. Cigarette smoke contains high concentrations of reactive oxygen species and tiny particles that are easily inhaled in the human body. ^18^ It is believed that smoking causes increased oxidative stress because of several mechanisms, including direct damage by radical species and the inflammatory response caused by cigarette smoking. The production of oxidative stress and reactive oxygene species due to the cigarette smoke is expected to increase VCAM-1 expression and decrease of e-NOS level. According to the previous research by Yang *et al* (2014), an increase of VCAM-1 expression in rat arteries after being exposed to cigarette smoke had been observed for seven days. ^19^ Translational research completed by Teasdale *et a*l (2014) and Pott *et al* (2017) also supported the findings that increased oxidative stress, reactive oxygene species, and VCAM-1 expression in endothelial cell cultures followed exposure to cigarette smoke. ^20^ Previously, researchers had been studying the influence of smoking on the levels of several biomarkers of oxidative stress, antioxidant status and redox status, including plasma hydroperoxides, e-NOS and VCAM-1. Using different assays to our study, they confirmed that smokers have elevated concentrations of VCAM-1 and compromised e-NOS status. ^21^

### Cigarette smoke extract induces expression of cell adhesion molecules

VCAM-1 is expressed in vascular endothelial cells, and expression of VCAM-1 may promote the adhesion of leukocytes to the endothelial cells. VCAM-1 accelerates the migration of adherent leukocytes along the endothelial surface, and promotes the proliferation of vascular smooth muscle cells; thus, VCAM-1 may play an essential role as a pro-atherogenic molecules. ^22^ Exposure to cigarette smoke in this study can increase VCAM-1 expression in the aorta although the increase is not statistically significant between the two groups. An insignificant increase in VCAM-1 expression was also found in the previous human research held by Noguchi (1999). In his previous research, soluble VCAM-1 levels were increased in smokers' serum but not significantly when compared to non-smokers’ serum. ^23^

Increase of VCAM-1 expression is a multifactorial process, smoking could not increase VCAM-1 independently without other risk factors such as dyslipidemia. Mu *et al* (2015) had proven this hypothesis by examining VCAM-1 expression in aortic tissue of dyslipidemia patients. As a result, VCAM-1 expression was positively correlated with triglyceride, total cholesterol and LDL levels while VCAM-1 and HDL had a negative correlation ^24^ because the expression of VCAM-1 in endothelial cells requires a trigger that is high lipid levels, especially LDL. An increase in oxidized LDL in the endothelium will be phagocytosed by macrophages. Recruitment of these macrophages requires the role of VCAM-1. ^25^ In our study, other factors contributing to the development of atherosclerosis such as dyslipidemia weren’t included. Our study did not use experimental animals with high-fat diets and serial lipid profile measurement Therefore, results of our study didn’t show any statistical significance of VCAM-1 expression between K (-) and K (+) groups.

### Cigarette smoke extract counteracts atheroprotective effects of endothelial nitric oxide synthase

Decreased bioavailability of NO is a central mechanism in the pathophysiology of endothelial dysfunction. Endhotelial nitric oxide synthetase (e-NOS) is an enzyme that resposible to produce NO in endothelial cells, so the level of eNOS can represent the availability of NO in endothelial cells. ^26^ Endothelial-cell dysfunction itself could be tested by acetylcholine response function and adenosine coronary flow reserve tests. ^27^ Celermajer *et al* (1992) published a study showing that smoking reduces flow-mediated dilatation (FMD) in systemic arteries in healthy young adults. ^28^

Our study showed that exposure to cigarette smoke can reduce levels of eNOS in the aorta. Our results are consistent with the findings of He *et al* (2017), which shows a significant decrease of eNOS level in endothelial cell cultures exposed to cigarette smoke. He *et al* (2017) showed that exposure to cigarette smoke in endothelial cell culture can reduce the expression of eNOS genes and proteins, resulting endothelial-cell dysfunction. ^29^ On the other hand, Su *et al* (1998) had already proven that administration of cigarette smoke extract can reduce the expression of genes and proteins eNOS. The effect of eNOS reduction depends on the duration of exposure to the cells. The longer the duration of cigarette smoke exposure, the more eNOS levels will be decreased. ^30^ In addition to decreasing eNOS at the gene level, Pini *et al* (2016) showed that exposure to secondhand smoke had also been shown to reduce eNOS at protein levels. eNOS levels decreased in the aorta of guinea pigs after exposure to cigarettes for eight weeks. ^31^

It has been demonstrated that smoking cigarettes triggers demethylation, leading to a consecutive reactivation of epigenetically silenced genes *in vitro* and *in vivo* of eNOS and NO production. ^32^ Peroxinitrites, a very reactive oxygene species and pro-oxidant properties from cigarette extract, is believed to promote demethylation and inactivation of e-NOS. ^33^ In addition, peroxynitrite and other free radicals can deactivate BH4 which is an important cofactor in eNOS production. This was explained by the research of Abdelghany *et al* (2018) which showed that exposure to cigarette smoke has been shown to reduce the BH4 cofactor and correlated with the amount of superoxide and NO production in endothelial cell cultures. ^34^ A decrease of e-NOS and NO level will increase vascular tone, increase expression of adhesion molecules, and trigger coagulation cascade and inflammation. ^35^ In the final pathway, smoking cigarettes leads to increase of aortic intima-medial thickness as an earlier sign of atherosclerosis

Based on this literature and our own data, we suggest that the exposure to cigarette smoke for 28 days daily might be an independent risk factor for atherogenic process through several mechanisms. Aortic IMT in this study increased in group K (+) as was also found in studies conducted by Ali *et al* (2012). ^36^ Increased aortic and entire blood vessels’ IMT are due to the pathological conditions such as apoptosis and excessive proliferation as a compensation mechanism. ^37^ In the previous study, the increase of IMT as a complication of endothelial dysfunction leads to the atherosclerosis process. ^38^ Cigarette smoke exposure underlies the endothelial dysfunction by the reduction of e-NOS level and increase of VCAM-1 expression. ^39^

Exposure to cigarette smoke also affects the histological structure of the aorta. In this study, we found not only an increase of IMT, but also structural changes marked by disorganization and vacuolization of smooth muscle cells in the tunica media of the aortic tissue. On the contrary, no changes were observed at the tunica intima level. Exposure to cigarette smoke for 28 days in the study of Ali *et al* (2012) also found the same results: no changes in the tunica intima were observed from the experimental rat. ^36^ Another experimental study from Jaldin *et al* (2013) found that exposure to cigarette smoke for eight weeks only resulted in a disorganization in the vascular smooth muscle cells in the tunica media. ^40^ Vacuolization is one of the complications from cytotoxic processes in the cells and an earlier marker of preclinical atherosclerosis. Chemical components from cigarette smoke can cause oxidative stress which is characterized by permanent vacuolization in cells. In the microscopic phenotyping, vacuolization makes the vascular smooth muscle cells have different shapes and sizes, thus resulting in the cells becoming disorganized and leading to atherosclerosis. ^41^

### Limitations

Every study has its limitations which emerge during the realization of the study, creating challenges and, thus, should be highlighted. Firstly, only 3 parameters are being studied which may limit the wide pathophysiology of how nicotine could make acute toxicity in animal model. Secondly, results from animal models often do not translate into replications in human models. Level of e-NOS and VCAM-1 expression in Wistar rats are typically transient, whereas in human they can persists for many years. Another crucial difference is IMT, which is usually much lower in the Wistar rats than in humans. These factors may have an impact on the interpretation of our results. Thus, the findings should be interpreted within the context of this study and its limitations. The strengths of the study were its high statistical power and the homogeneity of each group to enable comparisons between groups and periods.

## Conclusion

The present study indicates that smoking cigarettes adversely affects endothelial function and increases the risk of atherosclerosis. Smoking cigarettes as a risk factor for atherosclerosis is closely linked to the increased inflammatory process on the vascular endothelium. Low e-NOS level and high VCAM-1 level observed following smoke exposure may increase aortic IMT. Furthermore, smoking has also been found to influence the aortic IMT. Aortic IMT itself reflects the level of established CVD risk factors in apparently healthy men and women, adding to the evidence that cigarette smoking contributes to CVD through its inflammatory effects on the vascular endothelium.

### Ethic approval

Animal experimental study were conducted under the approval of the Institutional Animal Care and Use Committee of Universitas Airlangga (UNAIR), Surabaya, Indonesia (animal approval no: 2.KE.184.10.2019) under the name of Meity Ardiana as the Principal Investigator. Study was carried out in strict accordance to internationally-accepted standards of the Guide for the Care and Use of Laboratory Animals of the National Institute of Health.

## Data availability

### Underlying data

Figshare. Raw Data - Acute effects of cigarette smoke: https://doi.org/10.6084/m9.figshare.14252639.v1. ^42^

BioRxiv: Acute Effects of Cigarette on Endothelial Nitric Oxide Synthase, Vascular Cell Adhesion Molecule 1 and Aortic Intima Media Thickness “Cigarette smoke–induced pro-atherogenic changes”: https://doi.org/10.1101/2021.01.17.426972. ^43^

Universitas Airlangga Repository: http://repository.unair.ac.id/id/eprint/102952 (DOI 10.1101/2021.01.17.426972)

This project contains the following extended data: acute effect of jinten hitam (Nigella sativa) as protective factor from cigarette smoke on aortic intima media thickness of rat. ^44^

Data are available under the terms of the Creative Commons Attribution 4.0 International license (CC-BY 4.0).
