# Supplementary material for: Acute effects of cigarette smoke on Endothelial Nitric Oxide synthase, vascular cell adhesion molecule 1 and aortic intima media thickness
Source: F1000Res. 2023 Oct 10;10:396. Originally published 2021 May 18. [Version 4] doi: 10.12688/f1000research.28375.4 (PMC10690037; doi:10.12688/f1000research.28375.4)
Supplement: Peer review history

# Reviewer report: Reviewer response for version 4

**DOI:** 10.5256/f1000research.157098.r213800

**Published:** 2023-11-30

**Authors:** Leila Mohammadi

**Affiliations:** 1 Division of Cardiology, University of California San Francisco, San Francisco, California, USA

The current revised format looks good.

Is the work clearly and accurately presented and does it cite the current literature?

Yes

If applicable, is the statistical analysis and its interpretation appropriate?

I cannot comment. A qualified statistician is required.

Are all the source data underlying the results available to ensure full reproducibility?

Yes

Is the study design appropriate and is the work technically sound?

Yes

Are the conclusions drawn adequately supported by the results?

Yes

Are sufficient details of methods and analysis provided to allow replication by others?

Yes

Reviewer Expertise:

Cardiovascular disease, endothelial function, nitric oxide, cell culture, biomarkers of endothelial function, women cardiovascular health, tobacco cannabis, e-cigarettes use and cardiac health,

I confirm that I have read this submission and believe that I have an appropriate level of expertise to confirm that it is of an acceptable scientific standard.

# Reviewer report: Reviewer response for version 3

**DOI:** 10.5256/f1000research.151452.r182590

**Published:** 2023-10-05

**Authors:** Leila Mohammadi

**Affiliations:** 1 Division of Cardiology, University of California San Francisco, San Francisco, California, USA

I have thoroughly reviewed the manuscript by Ardiana et al. on the acute effects of cigarette smoke on endothelial NOS, vascular adhesion molecule 1, and aortic intima media thickness. While the manuscript is well written and the data are presented meticulously, I have some concerns about the study's design and novelty, which I believe need to be addressed for further clarity and scientific rigor. My specific concerns are as follows:

1. **Insufficient Explanation of Exposure Design**: The authors have not adequately explained the exposure session and puff volume, which are essential to ensure the relevance of the study to real-life smoking scenarios. I recommend adding these details to the experimental design and group section to provide a comprehensive understanding of the exposure conditions.
2. **High Number of Cigarettes Used**: It appears that the study involved the use of 40 or more cigarettes, indicating 2 or more packs a day. This exposure level seems to be an extreme amount of smoke inhalation, which may not accurately reflect real-life daily use. I urge the authors to clarify why such a substantial amount of exposure was chosen for this study, and if it aligns with the objectives and relevance to human smoking patterns.
3. **Correlations and sVCAM1 Levels**: While I appreciate the inclusion of correlations between different variables, it is essential to acknowledge that the impact of acute cigarette smoke on sVCAM1 levels has already been investigated in previous studies. The authors should provide a concise rationale for re-examining this aspect and highlight any novel findings or unique perspectives offered in their study.
4. **Clarification on Terminology**: The term "cigarette smoke extract" is used multiple times throughout the manuscript, but it is unclear whether the authors are referring to the actual extract or the smoke itself. I kindly request the authors to clarify this terminology to avoid confusion and ensure accuracy in the interpretation of their findings.

I believe addressing these concerns will significantly enhance the clarity and scientific value of the study. Please feel free to reach out if you require any further information or have any questions regarding my feedback.

Is the work clearly and accurately presented and does it cite the current literature?

Yes

If applicable, is the statistical analysis and its interpretation appropriate?

I cannot comment. A qualified statistician is required.

Are all the source data underlying the results available to ensure full reproducibility?

Yes

Is the study design appropriate and is the work technically sound?

Yes

Are the conclusions drawn adequately supported by the results?

Yes

Are sufficient details of methods and analysis provided to allow replication by others?

Yes

Reviewer Expertise:

Cardiovascular disease, endothelial function, nitric oxide, cell culture, biomarkers of endothelial function, women cardiovascular health, tobacco cannabis, e-cigarettes use and cardiac health,

I confirm that I have read this submission and believe that I have an appropriate level of expertise to confirm that it is of an acceptable scientific standard, however I have significant reservations, as outlined above.

## References

[1] : Circulating ICAM-1 and VCAM-1 in peripheral artery disease and hypercholesterolaemia: relationship to the location of atherosclerotic disease, smoking, and in the prediction of adverse events. *Thromb Haemost* .1998;79(6) :1080-5 9657427

[2] : Cigarette Smoke Extract: A Preclinical Model of Tobacco Dependence. *Curr Protoc Neurosci* .2016;77: 10.1002/cpns.14 9.54.1-9.54.10 10.1002/cpns.14 27696362PMC5113292

[3] : Structural and functional remodeling of the female Apoe-/- mouse aorta due to chronic cigarette smoke exposure. *Am J Physiol Heart Circ Physiol* .2021;320(6) : 10.1152/ajpheart.00893.2020 H2270-H2282 10.1152/ajpheart.00893.2020 33834870PMC8289362

[4] : Haemostatic parameters related to lipids and adhesion molecules. *Blood Coagul Fibrinolysis* .1999;10(8) : 10.1097/00001721-199912000-00003 465-70 10.1097/00001721-199912000-00003 10636457

[5] : The influence of tobacco smoking on adhesion molecule profiles. *Tob Induc Dis* .2002;1(1) : 10.1186/1617-9625-1-1-7 7-25 10.1186/1617-9625-1-1-7 19570245PMC2671531

# Response

**Published:** 2023-10-05

**Authors:** Ricardo Adrian Nugraha

**Affiliations:** Airlangga University, Indonesia

Thank you for reviewing our manuscript and give constructive feedback to improve our manuscript better for the reader. There are several points from the reviewer which we would like to address:

**1. Insufficient Explanation of Exposure Design**: The authors have not adequately explained the exposure session and puff volume, which are essential to ensure the relevance of the study to real-life smoking scenarios. I recommend adding these details to the experimental design and group section to provide a comprehensive understanding of the exposure conditions.

 Author Response: As it might be difficult to measure real-time cigarette smoke and nicotine concentrations at this moment, thus the level of cigarette smoke was titrated to the amount of total suspended particulate matter that was measured using a real-time monitoring probe (Casella Microdust Pro; Casella USA, Nashua, NH, USA). We want to study the relationship between acute inhalation of suspended particulate level and tobacco smoke concentration in the exposure room. We developed experimental models use of 40-puff smoking session conducted during the 12-h tobacco smoke exposure sessions each day for 28 days. Puff volume may be up to 100-140 mL. These experimental models were made using a research cigarette of conventional construction, made only from Virginia tobacco, which contains 39 mg of tar and 2.3 mg of nicotine. During the experiment, air was continuously drawn in parallel, through three resin-filled glass filters (XAD-4) set up in parallel at approximately 2 L per min. 

**2. High Number of Cigarettes Used**: It appears that the study involved the use of 40 or more cigarettes, indicating 2 or more packs a day. This exposure level seems to be an extreme amount of smoke inhalation, which may not accurately reflect real-life daily use. I urge the authors to clarify why such a substantial amount of exposure was chosen for this study, and if it aligns with the objectives and relevance to human smoking patterns.

 Author Response: Smoking behaviour is various among socioeconomic, education, occupation and geographic patterns. Indeed, the subsequent deposition of smoke constituents in the respiratory tract of smokers is an equally complex process both in terms of quantification of dose and time. In fact, assuming that the consumed portion of each cigarette contains 0.7 g of combustible material, 39 mg of tar, 2.3 mg of nicotine and that the smoker inhales two-thirds of the CO _2 _produced by the cigarette, smoking two packs a day would produce an error of about 0.6 mol of CO _2 _per day or 2-4% of true CO _2 _production. Thus, only a heavy smoking is expected to result in an acute toxicity within 28 days, whilst 20 cigarettes, indicating 1 packs a day might develop subacute toxicity within 1-2 years.

**3. Correlations and sVCAM1 Levels**: While I appreciate the inclusion of correlations between different variables, it is essential to acknowledge that the impact of acute cigarette smoke on sVCAM1 levels has already been investigated in previous studies. The authors should provide a concise rationale for re-examining this aspect and highlight any novel findings or unique perspectives offered in their study.

 Author Response: Yes, previous studies from Blann AD, et al (1998), Scott DA and Palmer RM (2002) had been studying the relationship of soluble intercellular adhesion molecule-1 (sICAM-1) and vascular cell adhesion molecule-1 (sVCAM-1) with smoking. The reason why we re-examine the role of cell adhesion molecules is because sVCAM-1 is the essential pro-inflammatory and pro-atherogenic proteins that represent a hallmark of endothelial dysfunction and atherosclerosis. We want to study the putative mechanism of subclinical atherosclerosis by short duration of tobacco smoke. Our results showed that acute exposure of tobacco smoke can enhance the upregulation of sVCAM-1 and the involvement of important known intermediaries which leading to subclinical atherosclerosis.  

**4. Clarification on Terminology**: The term "cigarette smoke extract" is used multiple times throughout the manuscript, but it is unclear whether the authors are referring to the actual extract or the smoke itself. I kindly request the authors to clarify this terminology to avoid confusion and ensure accuracy in the interpretation of their findings.

 Author Response: I apologize for that error. After careful discussion with all of the authors, it is not cigarette smoke extract but the smoke itself. We will revise it.

 Once again, thank you very much for the suggestion and feedbacks.

# Reviewer report: Reviewer response for version 2

**DOI:** 10.5256/f1000research.137198.r150339

**Published:** 2022-10-18

**Authors:** Amilia Aminuddin

**Affiliations:** 1 Department of Physiology, Faculty of Medicine, Universiti Kebangsaan Malaysia Medical Centre, Kuala Lumpur, Malaysia

The authors have made appropriate changes and there are no other queries from me.

Is the work clearly and accurately presented and does it cite the current literature?

Partly

If applicable, is the statistical analysis and its interpretation appropriate?

Partly

Are all the source data underlying the results available to ensure full reproducibility?

Yes

Is the study design appropriate and is the work technically sound?

Yes

Are the conclusions drawn adequately supported by the results?

Yes

Are sufficient details of methods and analysis provided to allow replication by others?

Yes

Reviewer Expertise:

Cardiovascular

I confirm that I have read this submission and believe that I have an appropriate level of expertise to confirm that it is of an acceptable scientific standard.

# Reviewer report: Reviewer response for version 1

**DOI:** 10.5256/f1000research.31386.r145923

**Published:** 2022-08-05

**Authors:** Amilia Aminuddin

**Affiliations:** 1 Department of Physiology, Faculty of Medicine, Universiti Kebangsaan Malaysia Medical Centre, Kuala Lumpur, Malaysia

This study determines the effects of 1-month exposure to tar and nicotine on eNOS, VCAM-1, and aortic stiffness in a rats model.

- The authors should highlight the novelty of this study since there are a lot of studies that have been conducted in this area and for these parameters. The parameters also were limited (three parameters only). If the authors can add more parameters then it will be good. 
- Out of 44 references, only 12 references are the latest (within 5 years).
- In the results section, the authors should show which statistical analysis has been conducted, rather than presenting both. For example, Table 2 presented both the t-test and Mann-Whitney, and actually one should be chosen based on the normality of the parameters. The mean value and SD in the table also should use a dot, instead of a comma. 
- There were abbreviations used without the full names given, such as TGF and BH4.
- Please identify rats as animals and not subjects.
- The manuscript should be proofread as there were grammatical/spelling errors.
- A sample size of nine per group is more than enough and not the limitation of the study.

Is the work clearly and accurately presented and does it cite the current literature?

Partly

If applicable, is the statistical analysis and its interpretation appropriate?

Partly

Are all the source data underlying the results available to ensure full reproducibility?

Yes

Is the study design appropriate and is the work technically sound?

Yes

Are the conclusions drawn adequately supported by the results?

Yes

Are sufficient details of methods and analysis provided to allow replication by others?

Yes

Reviewer Expertise:

Cardiovascular

I confirm that I have read this submission and believe that I have an appropriate level of expertise to confirm that it is of an acceptable scientific standard, however I have significant reservations, as outlined above.

# Response

**Published:** 2022-08-07

**Authors:** Ricardo Adrian Nugraha

**Affiliations:** Airlangga University, Indonesia

This study determines the effects of 1-month exposure to tar and nicotine on eNOS, VCAM-1, and aortic stiffness in a rats model.

- The authors should highlight the novelty of this study since there are a lot of studies that have been conducted in this area and for these parameters. The parameters also were limited (three parameters only). If the authors can add more parameters then it will be good. 

**Author Response: **The novelty of this study is the strong correlation between those three parameters in the setting of acute cigarette smoke exposure, which may change the paradigm about only chronic or sub-chronic cigarette smoke exposure can induce the atherogenic process. Increasing evidence suggests that cigarette smoke exposure, even in the short-term period could induce VCAM-1 (enhance pro-atherogenic properties), and a decrease in e-NOS level (anti-atherogenic depletion). Thus, acute cigarette smoke may represent a significant risk factor for atherosclerosis by increasing aortic IMT. This evidence is discussed herein.

  

- Out of 44 references, only 12 references are the latest (within 5 years).

**Author Response:** Thank you for your suggestion. We try to replace the reference with the latest one. However, several data and information are only available in the old references

  

- In the results section, the authors should show which statistical analysis has been conducted, rather than presenting both. For example, Table 2 presented both the t-test and Mann-Whitney, and actually one should be chosen based on the normality of the parameters. The mean value and SD in the table also should use a dot, instead of a comma. 

**Author Response: **Table 2 (IMT) and Table 3 (e-NOS) used Mann-Whitney u test. On the contrary, Table 4 (VCAM-1) used an independent t-test. This is based on the normality test by the Shapiro-Wilk test. Thank you for your constructive feedback to shape our manuscript better.

  

- There were abbreviations used without the full names given, such as TGF and BH4.

**Author Response:** Thank you for your suggestion, we have added TGF (transforming growth factor) and BH4 (Tetrahydrobipterin) in the abbreviation lists

  

- Please identify rats as animals and not subjects.

**Author Response: **Thank you for your suggestion, we have replaced “subjects” with “animals”

  

- The manuscript should be proofread as there were grammatical/spelling errors.

**Author Response:** Thank you for your constructive feedback. We try to fix the grammatical/spelling errors in the second version of the manuscript.

  

- A sample size of nine per group is more than enough and not the limitation of the study.

**Author Response:** Thank you for your suggestion, we have removed the sample size in the limitation of this study
